# MiRNA and Exosomal miRNA as New Biomarkers Useful to Phenotyping Severe Asthma

**DOI:** 10.3390/biom13101542

**Published:** 2023-10-18

**Authors:** Piera Soccio, Giorgia Moriondo, Donato Lacedonia, Pasquale Tondo, Dalila Pescatore, Carla Maria Irene Quarato, Mauro Carone, Maria Pia Foschino Barbaro, Giulia Scioscia

**Affiliations:** 1 Department of Medical and Surgical Sciences, University of Foggia, 71122 Foggia, Italy; giorgia.moriondo@unifg.it (G.M.); donato.lacedonia@unifg.it (D.L.); pasquale.tondo@unifg.it (P.T.); alilad.pes@gmail.com (D.P.); mariapia.foschino@unifg.it (M.P.F.B.); giulia.scioscia@unifg.it (G.S.); 2 Institute of Respiratory Diseases, Policlinico of Foggia, 71122 Foggia, Italy; carlamariairene.quarato@gmail.com; 3 UOC Pulmonology and Pulmonary Rehabilitation, Istituti Clinici Scientifici Maugeri IRCCS—Bari, 70124 Bari, Italy; mauro.carone@icsmaugeri.it

**Keywords:** asthma, biomarkers, exosome, microRNA, severe asthma

## Abstract

Severe asthma (SA) is a chronic inflammatory disease of the airways. Due to the extreme heterogeneity of symptoms, new biomarkers are currently needed. MiRNAs are non-coding RNAs that negatively regulate gene expression at the post-transcriptional level. In biological fluids, miRNAs are contained within exosomes, vesicles capable of giving miRNAs considerable stability and resistance to degradation by RNAses. The main function attributed to the exosomes is intercellular communication. The goal of our study was to analyze intracellular and exosomal miRNAs in order to demonstrate their potential use as non-invasive biomarkers of asthma by showing, in particular, their role in SA. We detected miRNAs by qRT-PCR in both serum and serum-derived-exosomes of asthmatic patients and healthy controls. The levels of almost all analyzed intracellular miRNAs (miR-21, miR-223, and let-7a) were greater in asthmatic patients vs. healthy control, except for miR-223. In detail, miR-21 was greater in SA, while let-7a increased in mild-to-moderate asthma. On the other hand, in exosomes, all analyzed miRNAs were higher in SA. This study identified a series of miRNAs involved in SA, highlighting their potential role in asthma development and progression. These results need validation on a larger cohort.

## 1. Introduction

Asthma is a chronic inflammatory disease characterized by bronchial obstruction and airway hyperreactivity (AHR) [1]. It is considered a heterogeneous disease sustained by multiple biological pathways that manifest with dyspnoea, wheezing, and cough [2]. The extreme heterogeneity of asthma has led to the identification of different phenotypes and endotypes of the disease, in which a specific pathogenetic mechanism corresponds to a biological framework and consequent clinical manifestations [2]. 

The characterization of the disease endotype mainly recognizes two mechanisms underlying the inflammatory process: (i) the activation of the type 2 immune response, implicated in allergic pathogenesis, whose activation leads to the release of specific pro-inflammatory cytokines and whose final mediator of inflammatory process is the eosinophilic granulocyte; and (ii) the type 1 immune response, which generally recognizes different inducing factors (pollutants, smoke, viruses, etc.) and which takes the form of a different activation of the cytokine pattern, with the neutrophil granulocyte as the ultimate effector [3]. Briefly, the “endotypes” of asthma can be classified as “type 2” and “non-type 2” according to the level of T helper 2 (Th2) and innate lymphoid (ILC2) cells responses. Interleukins such as IL-4, IL-5, and IL-13 are produced by Th2 CD4+ cells or ILC2 in the type 2 endotype. IL-4, IL-5, and IL-13 are responsible for many of the common cellular responses to asthma, including inflammatory cell infiltration, mediator release, and airway remodeling. 

Unlike CD4+ T cells, there is little information about the role of CD8+ T cells in asthma [4]. However, CD8+ T cells are responsible for the development of AHR caused by allergens and lung inflammation [4] and can also produce type-2 cytokines [5]. 

Canonically, activated CD8+ T cells produce type 1 inflammatory cytokines such as IFNγ and release cytotoxic molecules; however, sometimes they can also respond to asthma-associated type 2 activation signals [5]. Under inflammatory conditions, CD8+ T cells can be phenotypically skewed from IFNγ production towards the synthesis of type-2 cytokines (TC2) [5].

Additionally, the expression of various non-coding RNAs could affect the function of CD8+ T cells [4].

These mechanisms demonstrate the pivotal role of the inflammatory response to environmental and intrinsic stimuli in asthma [6].

Different definitions of severe asthma have been formulated over time; currently, the one that best meets the management needs of the asthmatic patient is an operational definition in which severe asthma is defined as asthma that has required maximal inhalation therapies in the last 12 months to be controlled (step 4 or 5 according to GINA guidelines: high doses of inhaled corticosteroids associated with long-lasting β2 agonists or leukotriene modifiers or theophylline), therapies with systemic corticosteroids for more than six months, or asthma that remains “uncontrolled” despite these therapies [7].

To date, there is a limited choice of anti-inflammatory and bronchodilator treatments for asthma and there is no cure available for this disease. Therefore, the diagnostic and therapeutic assessment of patients with severe asthma needs new biomarkers [8].

MicroRNAs (miRNAs) are small non-coding RNA molecules, formed by approximately 18-22 nucleotides, which negatively regulate gene expression at the post-transcriptional level. They act through the recognition of specific mRNA targets in order to determine their degradation or translation repression [9]. An abnormal regulation of gene expression by miRNAs has been associated with the development and progression of numerous lung diseases, a feature that makes miRNAs potential new biomarkers for a chronic and heterogeneous disease such as severe asthma [10]. 

To date, we know that most miRNAs are found within the cell, and many studies have demonstrated their potential as promising diagnostic, prognostic, and therapeutic biomarkers of asthma [11]. However, there is a large portion of miRNA capable of migrating outside the cell and which can be found in body fluids, the so-called circulating miRNAs [12].

Several studies have shown that approximately 90% of circulating miRNAs form complexes with proteins, including Ago2 (Argonaute 2), NPM 1 (nucleophosmin 1), and high-density lipoprotein (HDL) [13]. The remaining 10%, however, is secreted in exosomes [14], vesicles capable of conferring miRNAs considerable stability and resistance to degradation by endogenous RNases.

Exosomes are nanometric vesicles of endosomal origin whose presence has been demonstrated in all biological fluids. They are produced by various types of eukaryotic cells and contain proteins, lipids, and nucleic acids, including miRNAs. The main function attributed to exosomes is intercellular communication. In fact, it is thought that the cell that generates them is capable of influencing the behavior of the cell that receives them through the transfer of their content [15].

Exosomes are therefore involved in intracellular exchanges in both physiological and pathological conditions, and previous studies have shown an alteration of the composition of exosomes during various diseases [16,17], a characteristic that predisposes them to use as possible diagnostic biomarkers, predictors of the degree of disease activity and progression, and as potential tools for effective and decisive therapeutic strategies [18].

Emerging evidence shows that exosomes and, in particular, exosomal miRNAs released from asthma-associated cells, such as mast cells, eosinophils, neutrophils, and T lymphocytes, may function as mediators of information exchange, thereby contributing to AHR, airway inflammation, and airway remodeling [19,20].

Despite extensive investigations into the role of miRNAs in the development of asthma [21,22,23], to date, studies on the functioning of exosomal miRNAs in this disease are lacking [24]. Based on the above, we therefore believe that alterations of intracellular miRNAs and circulating miRNAs can probably be implicated in the etiopathogenesis of asthma and of severe asthma.

Therefore, to develop effective therapeutic strategies and accurate diagnostic methods for asthma, it is necessary to elucidate the potential mechanisms involved, the target genes regulated by these exosomal miRNAs, and the asthma-related signaling pathways controlled by these target genes.

In this regard, the main objective of this study was the identification of specific intracellular and circulating miRNAs, isolated from serum samples of subjects with severe asthma, mild to moderate asthma, and control subjects, in order to compare the expression of microRNAs in the various groups and identify specific miRNAs that could be used as non-invasive biomarkers to diagnose and characterize the different asthmatic pheno-endotypes and to predict response to treatment.

In detail, we evaluated a group of miRNAs (miR-21, miR-223, and Let-7a), which, according to literature, seem to play a role in the development and pathogenesis of various forms of asthma. In particular, recent asthma-specific studies have shown that these miRNAs are important contributors to asthma susceptibility, exacerbations, and treatment response [25,26,27].

These microRNAs were evaluated both in serum and in exosomes isolated from the serum of asthma patients and compared with a group of healthy subjects in order to better understand how these small non-coding RNAs might play a role in asthma pathology.

**Figure 1:**
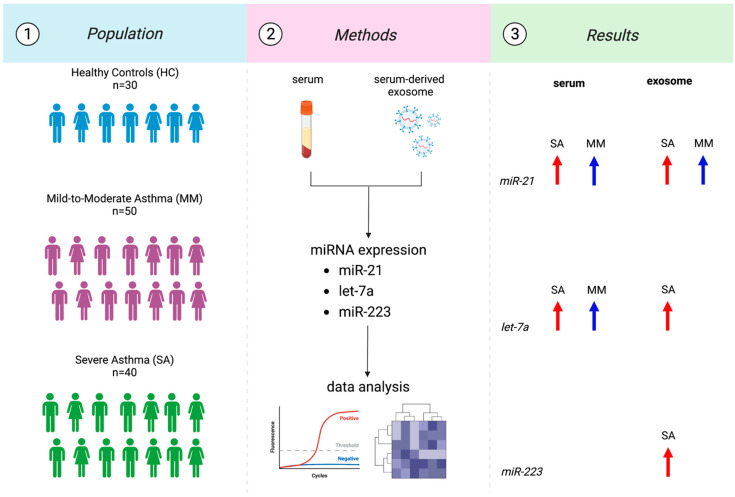
Flowchart of the study design.

The description of the study design with a graphical abstract is reported in Figure 1.

We measured the levels of several miRNAs (miR-21, miR-223, and let-7a) in serum and serum-derived exosomes from 30 healthy and 90 asthmatic patients with different phenotypes and severities. In detail, miR-21 increased either in SA (*p* < 0.0001) and MM (*p*: 0.0017) vs. HC. The same could be said for exosomal miR-21 (*p*: 0.0073; *p*: 0.0004). Let-7a increased in SA (*p*: 0.0009) and MM (*p* < 0.0001) compared to HC. Exosomal let-7a increased only in SA (*p* < 0.0001) vs. HC. Finally, miR-223 was upregulated just in exosome in SA (*p*: 0.0359). 

## 2. Materials and Methods

### 2.1. Study Population and Sampling

Patients with diagnosis of severe asthma (n = 40), patients with mild-to-moderate asthma (n = 50), and healthy subjects (n = 30) were consecutively enrolled through the outpatient clinic of asthma at the Institute of Respiratory Diseases, “Policlinico Riuniti” of Foggia, Italy. All the patients enrolled were European. 

Written informed consent was obtained from all study subjects and the study was approved by “Policlinico of Foggia” Ethical Committee (Institutional Review Board Approval n. 26/CE/2023). All protocols used comply with the principles of the Declaration of Helsinki. 

Asthma diagnosis was performed according to Global Initiative for Asthma (GINA) guidelines (GINA 2022 criteria; https://ginasthma.org/; accessed on 2 September 2022) [1]. Asthmatic patients were classified as having mild-to-moderate (MM) or severe asthma (SA) according to GINA 2022 criteria [1]. See Supplementary Methods for more details.

Exclusion criteria were as follows: smoking history, severe systemic comorbidities, treatment with immunomodulators or antihistamines, treatment with biologic therapy, no release of informed consent, or an upper respiratory tract infection during the last 4 weeks. 

All subjects enrolled underwent collection of demographic and clinical data, including functional variables using spirometry and inflammatory markers such as blood cell count and fractional exhaled nitric oxide (FeNO).

For all study participants, fasting serum was collected and stored at −80 °C until use. Briefly, peripheral venous blood samples were collected in EDTA-tubes and centrifuged at 3000× *g* for 10 min. 

### 2.2. Exosome Isolation

**Figure 2:**
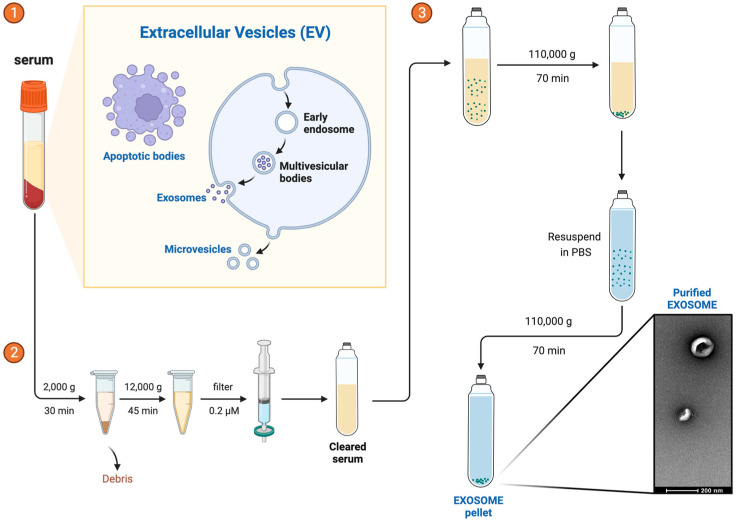
Exosome Isolation. Exosome isolation is a sequential process that involves steps ①, ②, and ③.

Exosomes were isolated from 250 µL of serum per patient by using serial centrifugation: 2000× *g* for 30 min to remove debris and thereafter 12,000× *g* for 45 min at 4 °C (Thermo Fisher Scientific, Waltham, MA, USA). The supernatant was filtered through a 0.22 µm filter (Corning^®^, Somerville, MA, USA), followed by an ultracentrifugation at 110,000× *g* for 70 min at 4 °C to pellet the exosomes (Beckman Coulter Optima L-90K Ultracentrifuge with Ti90 rotor; Beckman Coulter, Pasadena, CA, USA). The pellet was resuspended in 1 mL of PBS to wash it and ultracentrifuged again at 110,000× *g* for 70 min at 4 °C. The pellet was resuspended in 100 µL of PBS and frozen at −80 °C until use. All steps are described in Figure 2. 

### 2.3. Exosome Characterization by Western Blot 

Total protein concentration was determined using PierceTM BCA Protein Assay Kit, as recommended by the manufacturer (Thermo Fisher Scientific, Waltham, MA, USA). 

Then, 50 µg of exosome were separated on 4–20% Mini-PROTEAN^®^ TGX Stain-Free™ Protein Gels (Bio-Rad Laboratories–Inc., Hercules, CA, USA) and transferred to PDVF membranes (Invitrogen, Waltham, MA, USA). After blocking (Western Blocker^TM^ Solution, Sigma-Aldrich Co., St. Louis, MO, USA), membranes were incubated overnight at 4 °C with exosome anti-CD81 (Thermo Fisher Scientific, Waltham, MA, USA) and exosome anti-CD9 (Thermo Fisher Scientific, Waltham, MA, USA) primary antibodies (1:500 in Blocker^TM^ Solution). Anti-mouse (Bio-Rad Laboratories–Inc., Hercules, CA, USA) secondary antibodies (1:1000 in Blocker^TM^ Solution) were applied over 1 h under gentle agitation at room temperature before enhanced chemiluminescence revelation (ECL; Bio-Rad Laboratories–Inc., Hercules, CA, USA). 

### 2.4. Exosome Characterization by Multiplex Surface Marker Analysis

Analysis of surface protein expression on exosomes was performed using the MACSPlex Exosome kit human (Miltenyi Biotec, Cologne, Bergisch-Gladbach, Germany) following the manufacturers protocol. This kit enables the detection of 37 markers simultaneously and includes the two isotype controls (mIgG1 and REA control) corresponding to the antibodies. Briefly, 4–20 μg of protein or PBS as blank control were diluted in 120 μL MACSPlex buffer. The exosomes were incubated with 15 μL capture beads (containing the antibody-coated bead subsets) overnight at 4 °C under gentle agitation and without light. The exosome-bead complexes were washed using 1 mL MACSPlex buffer and centrifuged at 3000× *g* for 5 min at RT. The supernatant was aspirated and 5 μL of MACSPlex detection antibody mixture (CD9, CD63, and CD81 conjugated to APC) was added to the beads, then the samples were mixed by gentle vortexing and incubated for 1 h at RT under gentle agitation (450 rpm) and protected from light. 

Subsequently, 500 μL of MACSPlex Buffer was added to each tube and incubated in the dark for 15 min at RT in an orbital shaker (450 rpm). Samples containing exosomes were centrifuged and the supernatant was aspirated, leaving about 150 μL in the tube. Flow cytometric analysis was performed at Facs Canto II followed by Kaluza Analysis 2.1 (Beckman Coulter Life Sciences, Brea, CA, USA).

The concentration of the epitopes present on the exosome surface was obtained from the ratio between [beads + exosomes + Ab]/[beads + Ab] of the corresponding controls. 

### 2.5. Total RNA Extraction

For both sera and exosomes, total RNA was extracted by using TRIzol^TM^ Reagent (Thermo Fisher Scientific, Waltham, MA, USA) according to the manufacturer’s protocol. Concentration and quality of the eluted RNA were measured using NanoDrop™ 2000 Spectrophotometer (Thermo Fisher Scientific, Waltham, MA, USA). RNA purity was evaluated with the absorbance ratio A_260/280_.

### 2.6. RNA Reverse Transcription and miRNA’s Expression through qRT-PCR 

A 2 ng amount of total RNA was used for cDNA synthesis using TaqMan MicroRNA RT kit (Thermo Fisher Scientific, Waltham, MA, USA) according to the manufacturer’s protocol. Quantitative real-time polymerase chain reaction (qRT-PCR) assays for the quantitative determination of miRNA expression were performed in duplicate using ABI-PRISM 7300 Instrument (PE Applied Biosystems). miR-16 was used as endogenous control [28]. miRNA’s expression was calculated using the comparative 2^−∆∆Ct^ method [29]. MiRNA sequences used are listed in Table 1. 

### 2.7. Statistical Analysis

Sample Size Calculator was used to estimate sample size.

Comparisons between groups were performed by ANOVA or Kruskal–Wallis test depending on whether the data were normally distributed or not. Tukey test was used to carry out the post-hoc analysis. Data are presented as mean ± standard deviations (SD). To assess the distribution, a Shapiro–Wilk normality test was utilized. 

Spearman’s correlation was used to assess relationships between miRNAs expression levels and main respiratory data. 

Results were considered significant when *p*-values were ≤ 0.05. All statistical analyses were performed using GraphPad Prism software (version 9.0, GraphPad Software). Cluster analysis was performed on miRNAs’ expression value in order to identify their relationships using Orange (version 3.0, University of Ljubljana, Slovenia). See Supplementary Methods in Supplementary Materials for more details.

## 3. Results

### 3.1. Demographic and Clinical Characteristics of the Study Population

The demographic and clinical characteristics of study population are shown in Table 2. A total of 90 patients with different asthma severities (MM and SA), as well as 30 healthy controls, with similar age, body mass index (BMI), and sex ratio distributions, were enrolled in this study (Table 2).

Lung function was lower in SA patients. In detail, FEV1 and FEV1/FVC ratio were lower in SA. In addition, SA patients displayed higher levels of FeNO compared to MM patients (Table 2). 

### 3.2. Exosomes Characterization

**Figure 3:**
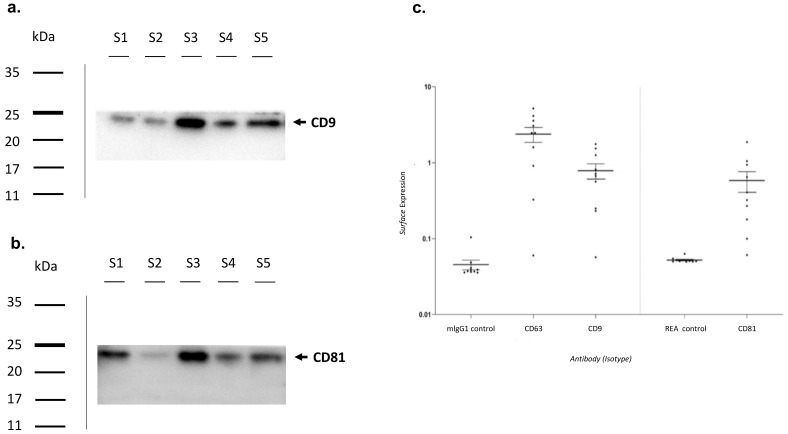
Exosomes characterization. Exosomes characterization by Western Blotting analysis using primary antibodies directed to (**a**) CD9—molecular weight 24 kDa and (**b**) CD81—molecular weight 22–26 kDa. Bands were obtained using an exposition of 100 s. (**c**) Exosomes characterization by Flow Cytometry. MlgG1 and REA were used as isotypic controls. MlgG1: Mouse Immunoglobulin G 1; REA: Recombinant Antibodies.

Characterization of exosomes was performed by Western Blot analysis (Figure 3). Briefly, three random samples were tested for the presence of CD9 and CD81, proteins characteristic of the exosomal surface. Blots were cut prior to hybridisation with antibodies during blotting. Full-length blots are presented in Supplementary Figure S1. Additional images of the Western Blot are included as Supplementary Materials. Both CD9 and CD81 were found in all samples analysed (Figure 3a,b).

Subsequently, a flow cytometric analysis was performed (Figure 3c). The surface expression of CD9, CD63, and CD81 was higher in almost all analyzed exosomes when compared to isotypic controls.

### 3.3. MiRNAs Expression

**Figure 4:**
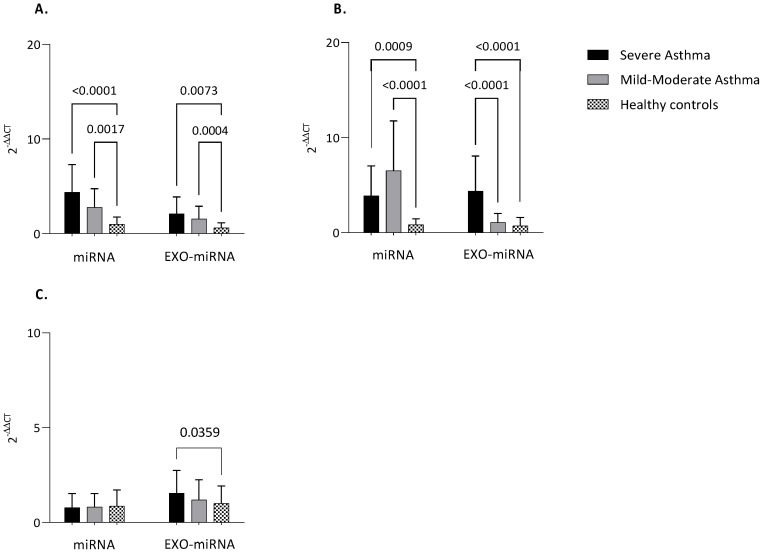
MiRNAs expression. Quantitative real-time PCR analysis of differentially expressed microRNAs in healthy subjects compared to patients with MA and SA both in serum and serum-derived exosomes. (**A**) miR-21; (**B**) Let−7a; (**C**) miR-223. MiR-16 was used as endogenous control. Comparisons between groups were performed by ANOVA. Data are reported as mean ± SD. Only statistically significant data are reported. Only if significant, the ANOVA *p*-value is reported. SA: Severe Asthma; MM: Mild-to-Moderate Asthma; exo-miRNA: exosomal-microRNA.

The expression of nearly all analyzed miRNAs, either intracellular or exosomal, was greater in asthmatic patients versus healthy controls (Figure 4). 

Notably, intracellular miR-21 was higher both in severe asthma and mild-to-moderate asthma compared to healthy controls (*p* < 0.0001; *p* = 0.0017). The same could be said about exosomal miR-21 (*p* = 0.0073; *p* = 0.0004) (Figure 4A).

The situation was completely different for Let-7a. Intracellular Le7-a was up-regulated both in severe and mild-to-moderate asthma compared to controls (*p* = 0.0009; *p* < 0.0001). On the other hand, exosomal Let-7a appears up-regulated in severe asthma compared mild-to-moderate and greater in severe asthma compared to healthy subjects (*p* < 0.0001; *p* < 0.0001) (Figure 4B). 

However, for miR-223 only a statistically significant difference in exosome was detected between severe asthma and control group (*p* = 0.0359). No difference was found in intracellular miR-223 (Figure 4C). 

**Figure 5:**
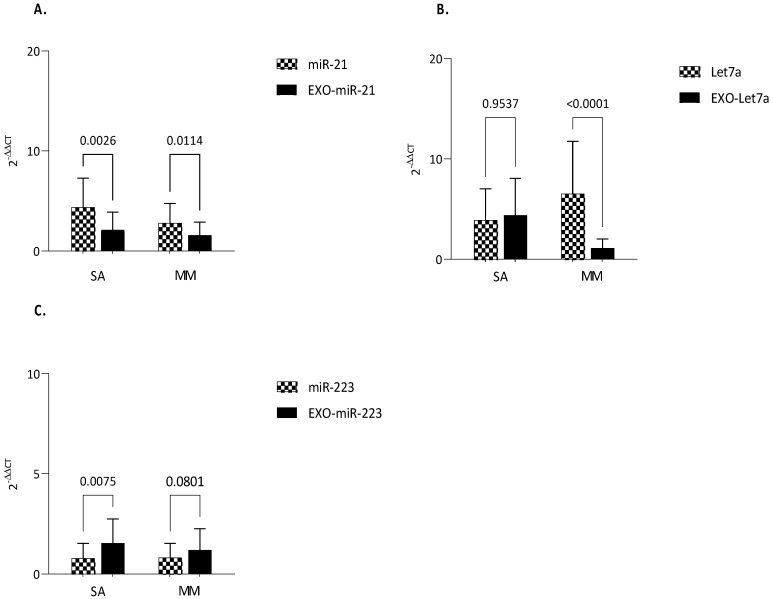
Comparison of miRNAs expression between serum and exosomes-derived serum. qRT-PCR analysis of microRNAs in patients with MA and SA either in serum and exosome-derived serum. (**A**) miR-21; (**B**) Let−7a; (**C**) miR-223. MiR-16 was used as endogenous control. Comparisons between groups were performed by ANOVA. Data are reported as mean ± SD. Only statistically significant data are reported. *p* ≤ 0.05 are significant. ANOVA *p*-value is reported. exo-miRNA: exosomal-microRNA.

Making an intracellular vs. exosomal comparison between severe and mild-to-moderate asthma, the following statistically significant differences were found: (i) the expression of intracellular miR-21, both in severe and mild-to-moderate asthmatics, was greater when compared with exosomal expression (*p* = 0.0026; *p* = 0.0114) (Figure 5A); (ii) exo-Let-7a was lower in mild-to-moderate asthma in comparison to intracellular Let-7a (*p* < 0.0001), while no difference was found between exo versus intracellular in severe asthma (Figure 5B); (iii) exo-miR-223 was up-regulated in both severe and mild-to-moderate asthma (*p* = 0.0075), but, in the latter, such a difference was not statistically significant (Figure 5C).

### 3.4. Clinical Correlations 

Correlation between miRNAs levels and main functional respiratory data (FEV1%, FVC%, FEV1/FVC ratio, and FeNO_50_) are listed in Table 3. 

In detail, exo-miR-21 correlated negatively with FEV1 and FEV1/FVC ratio. The same could be said for exosomal let-7a. Finally, exo-miR-223 correlated negatively with FEV1. 

### 3.5. Cluster Analysis 

**Figure 6:**
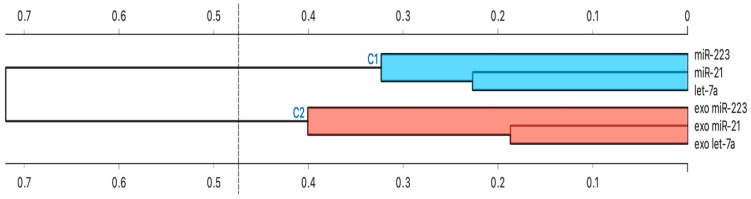
Hierarchical Cluster Analysis. The strength of correlation is inversely indicated by the black line’s length. Ward’s method was applied in cluster analysis. Distance level is plotted on the y-axis, individual units on the x-axis. Height ratio: 25% is shown.

Cluster analysis showed that all intracellular miRNAs had similar behavior. The same is true the same for exosomal miRNAs (Figure 6).

Briefly, we performed a hierarchical cluster analysis among all studied miRNAs to subdivide them according to their main characteristics (similarity and homogeneity).

## 4. Discussion 

It is known that miRNAs are emerging as important regulatory molecules that appear to be involved in the pathogenesis of several inflammatory diseases [30]. Often these diseases are particularly difficult to diagnose and characterize because the measurement of release mediators is unreliable [31]. Consequently, new non-invasive methods are needed to quantify inflammatory changes [32]. As a result, miRNA expression profiling in blood and other body fluids becomes important for the identification and development of novel non-invasive disease biomarkers.

Precisely with the aim of identifying new potential disease biomarkers, capable of characterizing the different asthmatic pheno-endotypes and defining the severity of the pathology, the present study has highlighted the importance of intracellular and/or exosomal microRNAs as hypothetical markers that could prove useful in clinical practice to study the best therapeutic strategy to implement depending on the cases of severe or mild-moderate asthma and to obtain correct management of the pathology.

For the study, we examined certain miRNAs in serum and serum-derived exosomes from 120 subjects. The subjects were divided on the basis of their clinical characteristics into: (i) people with severe asthma, (ii) people with mild to moderate asthma, (iii) control group. For all the microRNAs analysed, different patterns of expression were found between the three groups under examination both at serum and exosomal levels.

At the exosomal level, one of the molecules that, in our study, was upregulated in subjects with severe asthma compared to controls was miR-223. A miRNA involved in innate immunity, eosinophilic inflammation, and various obstructive airway diseases [33]. In recent years, several studies have shown elevated expression levels of miR-223 in both asthma and COPD [34].

Nevertheless, as mentioned above, our study seems to be true only at the exosomal level; in fact, no difference was found in the serum of our patients. Our data, in agreement with other sources in the literature [35,36], indicate that no difference in miR-223 expression was observed in serum between asthmatic patients and healthy controls.

Thus, despite its involvement in eosinophil development [37], airway inflammation [37], and airway resistance to airflow [38], in our study, the miR-223 in the serum among asthmatics and controls did not seem to be discriminated against. However, it could prove interesting at the exosomal level. Further studies will be needed to verify this hypothesis.

Another microRNA that showed a different pattern of expression between asthmatic patients and healthy controls was miR-21. Several studies have found an alteration of the expression of this miRNA in various inflammatory pathologies, demonstrating its involvement in the pathogenesis of asthma [39].

In particular, miR-21 has been shown to promote a type 2 immune response and to attenuate the type 1 immune response [40].

Furthermore, various studies have demonstrated that miR-21 is overexpressed in the airways of asthmatic subjects [41] and that its expression is able to regulate the production of IL-12 through the modulation of the IL-12/STAT4 in asthma [42]. Even the expression of IL-13, an interleukin produced by Th2 cells and responsible for the inflammation and symptoms associated with severe asthma, would seem to be favored by miR-21 [43].

At the exosomal level, previous studies conducted on mouse models have demonstrated the pro-inflammatory role of miR-21 contained in extracellular vesicles, observing that this miRNA is able to promote inflammation of the airways, oxidative stress, and the release of various proinflammatory cytokines [44].

In summary, in agreement with the data already present in the literature, in our study, the miR-21 resulted as up-regulated in both groups of asthmatic patients, both in serum and in exosomes isolated from serum, confirming the hypothesized pro-inflammatory effect of miR-21 and ranking as a new possible biomarker of asthma.

Therefore, although no significant difference in expression between severe and mild-moderate asthma was found in our study, we can consider this microRNA as a new, promising diagnostic biomarker useful for early discrimination of asthmatic patients.

Finally, our study brought to light new promising data on let-7a miRNA, highlighting a remarkable difference in let-7a expression both at the intracellular and exosomal levels between asthmatics and controls.

Let-7a expression is known to be altered in various pathological conditions, including cancer, Alzheimer’s disease, immune system diseases, allergic rhinitis, atopic dermatitis and, recently, its role in asthma has been demonstrated [45].

However, the hypothesized role of let-7a in asthma pathology is controversial. Numerous studies have hypothesized its possible anti-inflammatory role in asthmatic subjects [36,46], describing it as a key miRNA for the regulation of allergic inflammation in asthma. Conversely, other studies have highlighted the pro-inflammatory role of let-7a, demonstrating that the latter, together with other miRNAs such as miR-21, miR-142, and miR-146, promotes the inflammatory process underlying the asthmatic pathology [47].

The results obtained in our study therefore seem to support the latter option, agreeing with the pro-inflammatory role of let-7a in asthma, to the detriment of the previously hypothesized anti-inflammatory role.

In this regard, a recent study demonstrated that in vivo inhibition of let-7a can arrest the production of inflammatory cytokines by inhibiting the asthmatic phenotype [48].

Although there are not many references in this regard, in a recent study Zhang et al. studied the expression levels of three members of the let7 family (let-7a, let-7b, and let-7c) in the bronchoalveolar lavage (BAL) of asthmatic children, demonstrating an up-regulation of let-7a in asthmatic subjects compared to controls [47].

Furthermore, current research has highlighted that elevated levels of let-7a have been found in several patients with various inflammatory lung diseases [49].

Our data, therefore, agrees with what has been recently presented in the literature, underlining once again the pro-inflammatory role of let7-a and demonstrating that this miRNA seems to play an essential role in the inflammatory process typical of asthma. Furthermore, they provide further details about exosomal let-7a, which appears as an evident new possible biomarker of severe asthma that could lead to a new approach for the therapy of severe asthma.

Finally, in order to subdivide all the miRNAs analyzed according to their similarity and homogeneity, a cluster analysis was conducted using Ward’s hierarchical method. Following this analysis, we showed that all intracellular miRNAs were clustered together, while exosomal miRNAs were in a different cluster. In both clusters highlighted, however, there was an additional sub-clustering between miR-21 and let-7a. These results are consistent with the fact that miR-21 and let-7a in our study were up-regulated in both serum and serum exosomes isolated from asthmatic patients compared to healthy controls and highlight, once again, the pro -inflammatory of these two microRNAs.

These results were also consistent with the fact that miR-21 and let-7a negatively correlated with some pulmonary function parameters (FEV1 and FEV1/FVC).

Taken together, this evidence suggests that, in asthma, it is crucial to emphasize the significance of exosomal versus intracellular miRNAs. Specifically, our study found that some miRNAs are associated with asthma severity regardless of their source, while others have very specific effects depending on their origin (exosomal miR-223, for example). 

Thus, in asthmatic patients, cells release EVs in extracellular environments that mediate cell-to-cell communication and that, thanks to EVs-miRNA, are potentially involved in asthma development, progression, and severity. 

In addition, based on our results, lung function effects were only seen for exosomal miRNA, emphasizing once again that the source of miRNAs is essential in the context of asthma.

Future studies may be designed to deeply explore the content of extracellular vesicles and, in particular, of the miRNAs contained in them in asthmatic patients. Such efforts could provide insight into the role of EVs and EVs-miRNAs not only as useful biomarkers of disease, but also as regulators of asthma severity.

### Limitations of the Study

An extensive literature review is the basis for all the miRNAs studied. Mir-21, mir-223, and Let-7a were selected for their role in the development and pathogenesis of different forms of asthma. Recent asthma-specific findings show their role as significant contributor to the asthma process [20,21,22]. Further studies on more miRNAs will be required.

## 5. Conclusions 

This study demonstrated that microRNAs and especially exosomal microRNAs could be involved in asthma and could, in the future, develop a diagnostic potential for asthmatic pathology. In particular, it shown that the severity of asthma differently affects the expression of mir-21 and Let-7a either in serum or exosome. 

In light of the current evidence and the different expression patterns that these miRNAs have shown in different asthmatic pheno-endotypes, we believe that miRNAs could be used for anti-inflammatory therapies in the future. Non-invasive diagnosis and status monitoring of asthmatic subjects are essential for a chronic and heterogeneous disease such as asthma. However, for these small molecules to return to clinical practice, to date, there is still a long way to go. Future studies could allow the development of a diagnostic panel of miRNAs and/or exosomal miRNAs for asthma and for severe asthma in order to allow early diagnosis of the disease and pheno-endotyping of asthmatic patients, saving time and cost to clinical practice. Further studies will be needed to evaluate miRNAs in other biological samples, such as condensed exhaled breath or induced sputum, in order to further minimize the invasiveness of the technique. All these approaches, in our view, hold strong potential.

In conclusion, we believe that the study of miRNAs and exosomal microRNAs by molecular diagnostic techniques can bring great improvements for the non-invasive diagnosis and monitoring of numerous diseases such as asthma.

In asthma and, as shown in this study, in severe asthma in particular, the potential of circulating intracellular microRNAs is high, but numerous studies will be needed in the future to validate their clinical reproducibility on a large scale.

### Take Home Message 

Intracellular and exosomal miRNAs appear to be promising non-invasive biomarkers of asthma and their expression is strongly dependent on the severity of asthma (severe or mild-to-moderate).

## Figures and Tables

**Table 1:** miRNAs sequence.

Assay Name:	Assay ID	Species	miRNA Sequence
hsa-miR-16-5p	000391	human	UAGCAGCACGUAAAUAUUGGCG
hsa-miR-21-5p	000397	human	UAGCUUAUCAGACUGAUGUUGA
hsa-let-7a-5p	000377	human	UGAGGUAGUAGGUUGUAUAGUU
hsa-miR-223-5p	002098	human	CGUGUAUUUGACAAGCUGAGUU

**Table 2:** Demographic and clinical data of patients.

	ALL n = 120	HC n = 30	MM n = 50	SA n = 40	*p*-Value	TUKEYP OST-HOC
*Demographic data*						
sex, M/F	43/77	16/14	17/33	9/31	**0.0248** *	SA > HC = MM
age, years	53 ± 13.0	50 ± 9.7	56 ± 15.7	56 ± 10.6	0.1976	-
BMI, kg/m^2^	29 ± 6.7	27 ± 5.4	30 ± 6.5	29 ± 7.2	0.4689	-
*Lung function parameters*						
FEV1, %	85 ± 20.0	96 ± 20.3	86 ± 16.5	78 ± 20.8	**0.0046** *	HC > MM > SA
FEV1/FVC, %	76 ± 12.9	81 ± 6.0	80 ± 11.9	69 ± 13.6	<**0.0001** **	HC = MM > SA
FVC, %	99 ± 17.5	93 ± 17.4	100 ± 15.3	101 ± 15.0	0.2387	-
*Asthma biomarkers*						
FeNO_50_, ppb	27 ± 35.1	9.9 ± 3.5	25 ± 20.8	28 ± 41.1	0.5546	-
Blood eosinophils, % total cells	14.7 ± 42.8	1.9 ± 0.7	4 ± 3.2	11 ± 27.5	0.6637	-

HC: healthy control, MM: mild-to-moderate asthma, SA: severe asthma, FEV1: forced expiratory volume in 1st second, FVC: forced vital capacity, FeNO: fractional exhaled nitric oxide. *p* ≤ 0.05 are highlighted in bold. * *p* < 0.05; ** *p* < 0.001. Data are reported as mean ± SD and were analyzed using ANOVA test followed by Tukey post-hoc test.

**Table 3:** Correlation with main respiratory data.

Heading	Heading	FEV1, %	FVC, %	FEV1/FVC, %	FeNO_50_, %
*miR-21*	*serum*	−0.1433	0.0714	0.0359	0.0285
	*exosome*	−**0.1139** *	0.1478	−**0.1551** *	0.0893
*let-7a*	*serum*	−0.0344	−0.1773	−0.2152	0.0623
	*exosome*	−**0.2418** **	−0.1165	−**0.3680** **	0.1039
*miR-223*	*serum*	0.0536	0.1006	0.1590	−0.0133
	*exosome*	−**0.0004** *	−0.0131	−0.0780	−0.0827

FEV1: forced expiratory volume in 1st second, FVC: forced vital capacity, FeNO50: fractional exhaled nitric oxide > 50 ppb. R value is reported. *p*-value ≤ 0.05 is marked in bold. * *p* < 0.05; ** *p* < 0.001.

## Data Availability

Data are available upon request to Soccio Piera (piera.soccio@unifg.it).

## Supplementary Materials

The following supporting information can be downloaded at: https://www.mdpi.com/article/10.3390/biom13101542/s1, Figure S1: Characterization of exosomes by Western Blotting. Original and unprocessed western blot image. Bands were obtained using an exposition of 25s. Blots were cut prior to hybridisation with antibodies during blotting. (**a**) CD9-molecular weight 24 kDa and (**b**) CD81-molecular weight 22–26 kDa.

## Abbreviations

FeNO_50_: fractional exhaled nitric oxide >50 ppb; FEV1: forced expiratory volume in 1st second; FVC: forced vital capacity; HC: healthy control; miRNAs: microRNAs; MM: mild-to-moderate asthma; SA: severe asthma.
