# Supplementary material for: MiRNA and Exosomal miRNA as New Biomarkers Useful to Phenotyping Severe Asthma
Source: Biomolecules. 2023 Oct 18;13(10):1542. doi: 10.3390/biom13101542 (PMC10605226; doi:10.3390/biom13101542)
Supplement: Supplementary file 1 [file biomolecules-13-01542-s001.zip › biomolecules-2644918-supplementary.pdf]

SUPPLEMENTARY

Study Population and Sampling

In detail, MM was defined as asthma well controlled with Step 1 or 2 treatment: using short-acting beta agonists (SABA) as needed, low dose of ICS as maintenance controller treatment and/or low dose of ICS-LABA as needed, and/or antileukotrienes (LTRA). On the other hand, SA was defined as asthma in Step 3–5 treatment: at least medium/high dose of ICS-LABA maintenance controller treatment [5].

Statistical analysis

Generally, miRNAs with similar expression profiles are involved in the same cellular pathways and cluster together. In this regard, we used Ward's method, from which we obtained a plot in which the distance level is shown on the y axis, while the single units are shown on the x axis. In detail, Ward’s method makes it possible to calculate the deviations associated with all possible clusters and performs an aggregation which gives rise to the group having the minimum deviation. The distance between groups is the difference between the overall deviance and the sum of the deviances within the two groups.

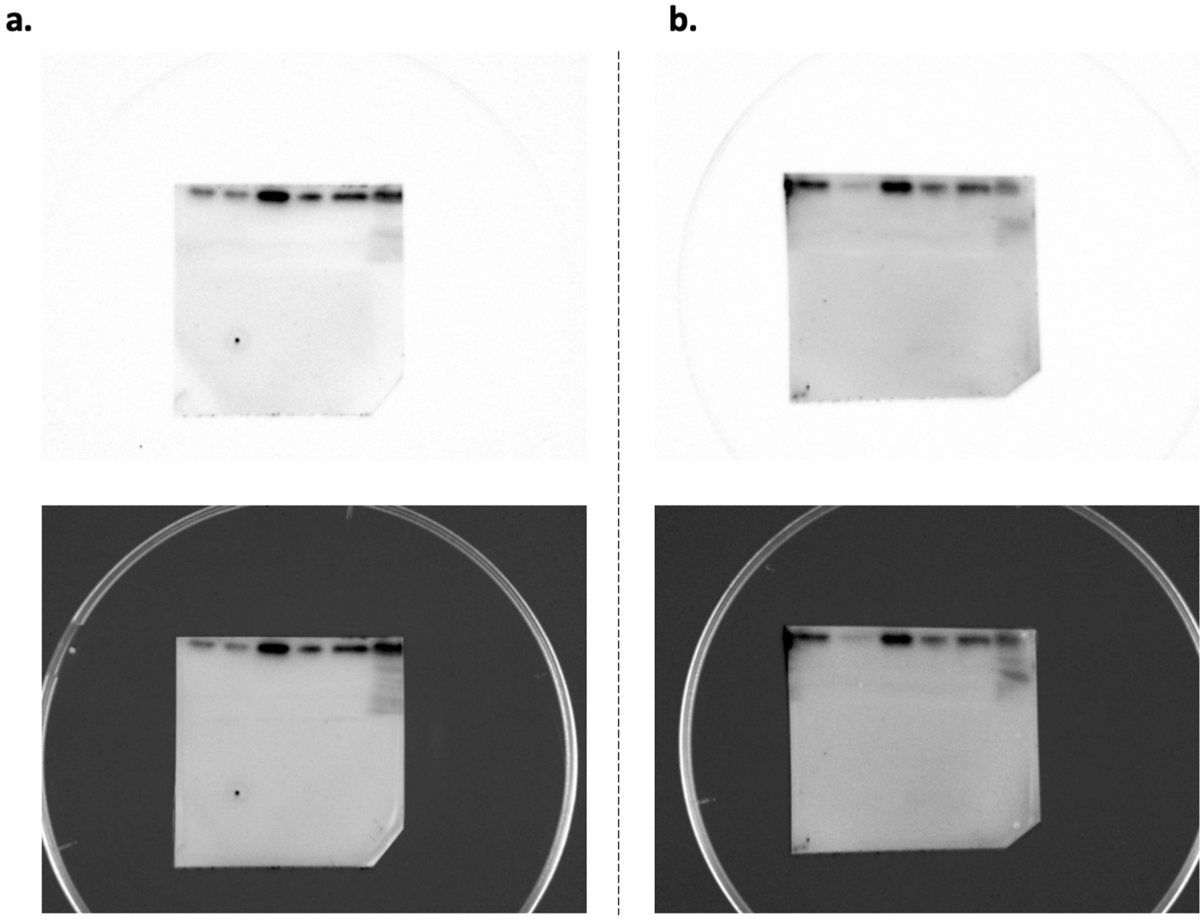

Supplementary Figure S1: Characterization of exosomes by Western Blotting. Original and unprocessed western blot image. Bands were obtained using an exposition of 25s. Blots were cut prior to hybridisation with antibodies during blotting. (**a**) CD9 - molecular weight 24 kDa and (**b**) CD81 - molecular weight 22–26 kDa.
